# Epidemiological Features of Leptospirosis and Identification of *Leptospira wolffii* as a Persistently Prevailing Species in North–Central Bangladesh

**DOI:** 10.3390/idr16040049

**Published:** 2024-07-23

**Authors:** Monira Sultana, Shyamal Kumar Paul, Syeda Anjuman Nasreen, Nazia Haque, Md. Kamrul Hasan, Arup Islam, Sultana Shabnam Nila, Afsana Jahan, Fardousi Akter Sathi, Tasmia Hossain, Syeda Jannatul Ferdaus, Meiji Soe Aung, Nobumichi Kobayashi

**Affiliations:** 1 Department of Microbiology, Nilphamari Medical College, Nilphamari 5300, Bangladesh; drmonira50@gmail.com; 2 Netrokona Medical College, Netrokona 2400, Bangladesh; drshyamal10@yahoo.com; 3 Department of Microbiology, Mymensingh Medical College, Mymensingh 2200, Bangladesh; nasreenm19@gmail.com (S.A.N.); drnaziahaque@gmail.com (N.H.); dr.arup@gmail.com (A.I.); nila1081@gmail.com (S.S.N.); drsathi.dmc@gmail.com (F.A.S.); doc.tasmia.hossain@gmail.com (T.H.); 4 Department of Ophthalmology, Sirajganj 250 Bed Bongamata Sheikh Fazilatunnesa Mujib General Hospital, Sirajganj 6700, Bangladesh; drkh.razib86@gmail.com; 5 Department of Microbiology, Pabna Medical College, Pabna 6602, Bangladesh; a.j.suravi@gmail.com; 6 Department of Oral Microbiology, Mymensingh Medical College, Mymensingh 2200, Bangladesh; jannat33bcs@gmail.com; 7 Department of Hygiene, School of Medicine, Sapporo Medical University, Sapporo 060-8556, Japan; meijisoeaung@sapmed.ac.jp

**Keywords:** leptospirosis, IgM LAT, IgM ELISA, nested PCR, *Leptospira wolffii*, Bangladesh

## Abstract

Leptospirosis is considered to be the most widespread, yet neglected, re-emerging zoonotic disease caused by infection with a pathogenic species of the genus *Leptospira*. Although this disease is prevalent in Bangladesh, the recent epidemiological status has not yet been well documented. In this study, we aimed to determine the prevalence of leptospirosis among febrile patients using different diagnostic methods and to characterize the epidemiological features and species of *Leptospira* in Mymensingh, north–central Bangladesh. Among the blood samples of 186 patients with suspected leptospirosis who met the inclusion criteria, including having a fever for more than 5 days (November 2021–June 2022), 88 samples (47%) were *Leptospira*-positive according to IgM LAT, IgM ELISA, or nested PCR (positivity rates: 38%, 37%, and 42%, respectively). Nested PCR showed a significantly higher positivity rate (54%) in patients with a short fever (5–10 day) than the other methods did, with lower rates among those with a longer fever. Leptospirosis cases were more common in males (68%), those 16–45 years of age (70%), residents of rural areas (81%), and farmers (41%). In addition to a fever, myalgia and jaundice were found in more than 70% of the patients, while variable symptoms were observed. The 16S rRNA sequencing analysis revealed that the *Leptospira* species in all the 22 samples tested were *L. wolffii*, belonging to the pathogenic subclade P2. This study showed the recent epidemiological features of leptospirosis in Bangladesh, indicating the presumptive predominance of *L. wolffii* since 2019.

## 1. Introduction

Leptospirosis is one of the most widespread zoonotic diseases globally, with a higher incidence in the tropics and subtropics, infecting more than a million people and causing approximately 60,000 deaths annually [1,2]. It is a waterborne disease transmitted by contact with the urine of animals or environments contaminated with pathogenic species of *Leptospira*. This zoonosis is considered to be a re-emerging disease due to increased contact between humans and animals via cultivation, urbanization, and poor sanitation in developing countries, which are associated with climate change [2,3]. In urban areas, rodents, particularly rats, are the main reservoirs, while in rural and agricultural areas, mammals such as cattle, pigs, dogs, and goats are potential hosts.

The clinical manifestation of leptospirosis is highly variable, and its signs and symptoms mimic several other diseases, making its diagnosis difficult. While anicteric leptospirosis is the milder form found in 90% of cases, the icteric form (Weil’s disease) is a severe disease that occurs in 5–10% patients, with a 5–40% mortality rate [4]. The disease onset is sudden and characterized by a fever, along with a consistent headache. The common symptoms include pain in the lower limbs, as well as other parts of the body, and a transient macular, maculopapular, erythematous, purpuric, or urticarial rash, usually on the trunk. Anorexia, nausea, and vomiting, along with constipation or diarrhea, are the other markers of anicteric leptospirosis. Weil’s disease is characterized by jaundice, renal failure, and hemorrhage, with a variable clinical course [5]. Though most cases of leptospirosis are mild and resolve spontaneously, penicillins and tetracyclines are used for antimicrobial therapy.

*Leptospira* is a Gram-negative, aerobic spirochete, comprising at least 72 species [6,7], which are genetically classified into two major groups, i.e., pathogenic and saprophytic clades. The pathogenic clade is divided into the P1 and P2 subclades [8], which have previously been referred to as the pathogenic and intermediate/host-mediated pathogenic groups, respectively [2,9]. The saprophytic group is also differentiated into two subclades (S1 and S2) [6,8]. Serologically, *Leptospira* is classified into more than 30 serogroups and 300 serovars based on the structure of the lipopolysaccharides in the outer membrane [7,10]. The P1 subclade accounts for the major etiologic agent of leptospirosis and comprises 20 species, including the globally distributed *L. interrogans*, *L. borgpetersenii*, and *L. krischneri* [6,11]. In contrast, the P2 subclade is relatively minor, yet genetically more divergent, including 21 species.

Although leptospirosis is prevalent in Bangladesh, limited information on its epidemiological features is available. An early study conducted in 1992 showed that 38% of suspected patients living in a rural flood-prone district were serologically positive [12]. During an outbreak of dengue fever in 2000, 18% of dengue-negative febrile patients in Dhaka tested positive for leptospirosis using PCR [13]. In 2001, leptospirosis was present in 8.4% of febrile people in a low-income urban community [14]. More recently, *Leptospira* was found in 17.6% (2018) and 35.7% (2019) of blood samples from suspected patients in a hospital in Mymensingh, north–central Bangladesh, using nested PCR [15,16]. In these studies, the sequence analysis of 16S rRNA revealed a major species, *L. interrogans*, in 2018; however, this was *L. wolffii* in 2019. Another study conducted in three places just before the COVID-19 pandemic described a low prevalence of leptospirosis (2%) among patients with acute febrile illness [17]. These findings suggest that the prevalence of leptospirosis might be changing and variable depending on the study site/population, although this disease is not uncommon in this country. Nevertheless, there is a dearth of information on the recent prevalence, the clinical characteristics of leptospirosis, and the prevailing species of *Leptospira*. This study was conducted to delineate the current epidemiological features of leptospirosis in Mymensingh, Bangladesh, following our previous studies in 2018–2019 [15,16].

## 2. Materials and Methods

This was a cross-sectional, observational study. Venous blood samples (5 mL) were collected aseptically, following the universal safety precaution, from febrile patients who visited Mymensingh Medical College Hospital and agreed to participate in this study for an eight-month period from November 2021 to June 2022. The inclusion criteria for the patients were as follows: (1) having had a fever for more than five days, with or without malaise, a headache, a rash, arthralgia, anorexia, abdominal pain, vomiting, conjunctival suffusion, or mental depression; (2) febrile patients having had any of the following symptoms: hepatic involvement, such as jaundice, renal involvement, such as oliguria, hematuria or pulmonary symptoms, such as a cough, dyspnea, hemoptysis, or respiratory distress syndrome; (3) febrile patients who did not have other diseases such as malaria, visceral leishmaniasis, dengue fever, and typhoid fever; and (4) patients who provided written informed consent. The exclusion criteria were as follows: (1) patients who refused to participate in this study, and (2) patients suffering from any febrile illness where any diseases other than leptospiral illness had already been diagnosed.

The whole-blood samples were left at room temperature for 30 min for clots to form, followed by centrifugation to obtain serum samples. These were used for further tests to screen leptospirosis using three methods, i.e., (1) latex agglutination test (IgM LAT); (2) IgM ELISA; and (3) nested PCR. IgM LAT and IgM ELISA were employed to detect *Leptospira*-specific serum IgM, which is detectable from day 5 onwards after the onset of symptoms and usually persists for 9–11 months.

For IgM LAT, the Leptorapide^®^ test (Linnodee Diagnostics Ltd., Ballyclare, Northern Ireland), a one-step agglutination assay, was used. Equal volumes of the serum samples and antigen-bound latex beads were mixed on a test board. As evidence of the antibody in the sera, visible agglutination occurred within 2–3 min of the gentle rotation of the card and was interpreted according to the score card provided with the kit.

For IgM ELISA, the *Leptospira* IgM ELISA kit (DRG international, Springfield, NJ, USA) was used according to the manufacturer’s indications. The microtiter strip wells of this kit were coated with purified *Leptospira* Patoc 1 antigen. Following the initial incubation of the serum samples in the wells, an enzyme conjugate and a chromogen (tetramethylbenzidine) were added successively, and we washed the wells before adding the reagents. When the serum samples contained IgM, the reaction mixture in the test well turned blue due to the catalysis of the chromogen with peroxidase, and then it turned to yellow after the addition of the stop solution. The reaction was read visually or with an ELISA reader.

Nested PCR was performed by using the protocol and primers for the detection of genus *Leptospira* as described previously [18]. In this PCR, the 16S rRNA of *Leptospira,* including the pathogenic and saprophytic clades, were targeted. Genomic DNA was extracted from the serum samples using the phenol–chloroform method. As a thermostable DNA polymerase, TaKaRa Ex Taq^®^ (Takara, BIO INC, Kusatsu, Japan) was used for the PCR. The second-round PCR product was electrophoresed on agarose gel and visualized as a band using a gel documentation system. To identify the *Leptspira* species, the nucleotide sequence of the partial 16S rRNA was determined using Sanger sequencing with the PCR product (289 bp) on an automated sequencer (Applied Biosystems^®^ 3500 Genetic Analyzer) at the National Institute of Biotechnology, Savar, Bangladesh (www.nib.gov.bd, accessed on 15 March 2023). The obtained sequence data were analyzed using BLAST, which is available on the NCBI website (http://blast.ncbi.nih.gov/Blast.cgi, accessed on 20 March 2024); one can search for the most similar sequence in the GenBank database. A phylogenetic tree of the 16S rRNA was constructed with the maximum likelihood method using the MEGA.6 software package. The multiple alignment of the nucleotides and amino acids was performed by using the Clustal Omega program (https://www.ebi.ac.uk/Tools/msa/clustalo, accessed on 10 April 2024). The sequence data of the representative *Leptospira* samples were deposited in GenBank under the accession numbers PP499264-PP499269 and PP499287. To discriminate the pathogenic and non-pathogenic groups, the nested-PCR products from the 16S rRNA were further used for restriction fragment length polymorphism (RFLP) analysis with ApoI, which digests PCR products from “non-pathogenic“ species as described previously [18]. In addition, conventional PCR was attempted to amplify the pathogenic species by using Lig1/Lig2 primers as described previously [19].

The sociodemographic factors and clinical symptoms of the individual patients were recorded on a data sheet, together with the laboratory test results. The difference in the variables between the *Leptospira*-positive and -negative patients was statistically analyzed with Fisher’s exact test using the js-STAR XR ver.1.1.9 software (https://www.kisnet.or.jp/nappa/software/star/index.htm, accessed on 20 April 2024). The difference in the *Leptospira*-positive rates using the different methods was analyzed with McNemar’s test. A *p* value < 0.05 was considered to be statistically significant.

## 3. Results

**Figure 1:**
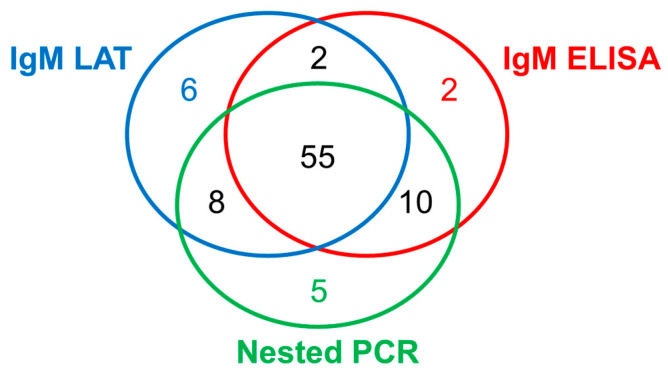
Number of human leptospirosis-positive samples diagnosed using the three methods (IgM LAT, IgM ELISA, and nested PCR).

During the study period, 186 samples were collected from febrile patients. Among them, the *Leptospira*-specific IgM was positive in 71 samples (38%) using LAT and 69 samples (37%) using ELISA, while leptospiral 16S rRNA was detected in 78 samples (42%) using nested PCR. Though 55 samples (30%) were *Leptospira*-positive when using all three methods, 2–10 samples showed positive results using only one or two methods (Figure 1). Among the 69 ELISA-positive samples, 67 samples (97%) were also positive according to LAT and/or PCR. In this study, the 88 samples (47%, 88/186) showing positivity when we used any of the three methods were judged as having leptospirosis.

**Figure 2:**
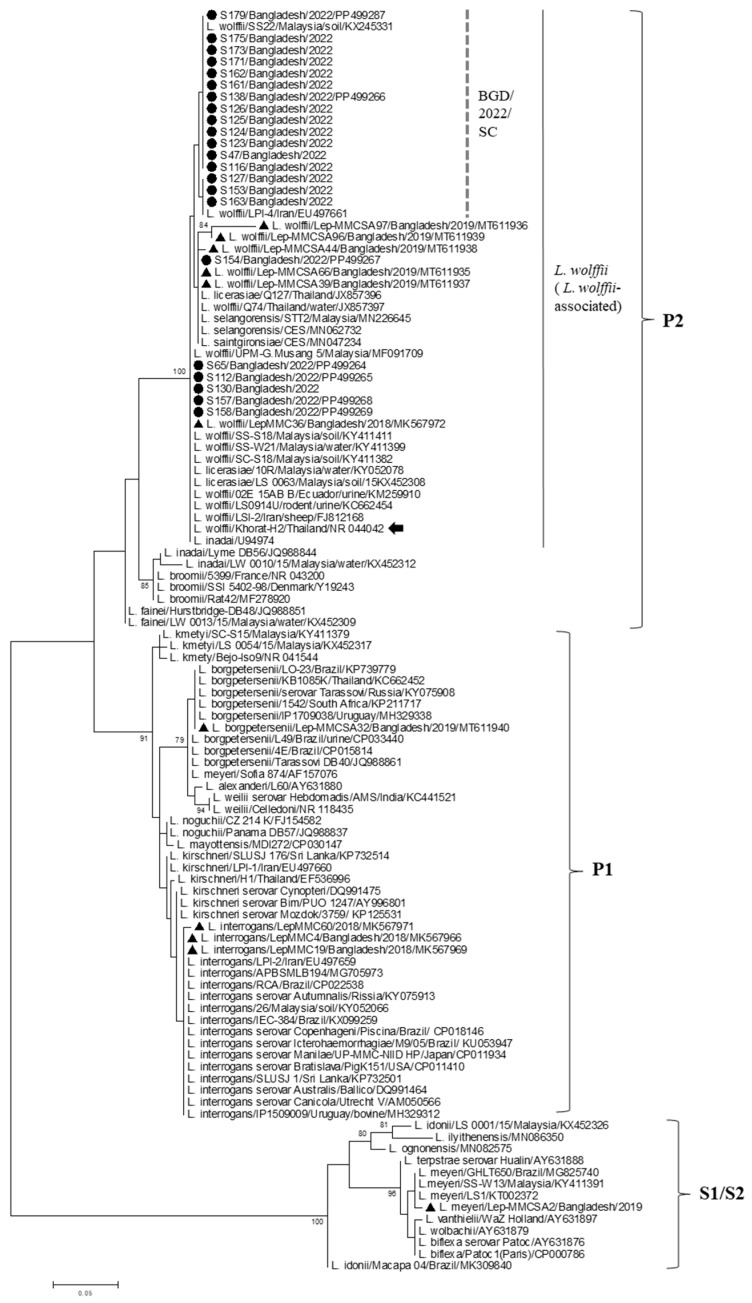
A phylogenetic dendrogram based on the partial 16S rRNA gene sequences of *Leptospira* constructed using the maximum likelihood method using the MEGA.6 program, following alignment with the ClustalW algorithm. The tree was statistically supported by bootstrapping with 1000 replicates, and the phylogenetic distances were measured using the Kimura 2-parameter model with uniform rates among the sites. The samples analyzed in this study are marked with closed circles, while those of a previous study in Mymensingh, Bangladesh, are denoted by triangles. An arrow shows the *L. wolffii*-type strain Khorat-H2. Bootstrap values of more than 80% are shown. The scale bar represents the genetic distance, i.e., the number of substitution per site. The subclades (P1, P2, and S1/S2) designated previously [8,11] are shown on the right. The vertical line shows a cluster of *L. wolffii* and species closely related to *L. wolffii* in the P2 subclade, while the dotted vertical line denotes a subcluster of *L. wolffii* in Bangladesh (BGD/2022/SC) identified in this study.

Through PCR-RFLP analysis of the nested PCR-positive samples, all 78 samples were assigned to “pathogenic” or “intermediate” species (i.e., not non-pathogenic group), while they were all negative according to PCR with Lig1/Lig2 primers specific to “pathogenic” *Leptospira*. These results showed that *Leptospira* in all the samples belonged to the “intermediate” species. The nucleotide sequence of the partial 16S rRNA was determined for 22 samples, which were selected from the specimens showing clear and prominent PCR products on agarose gel electrophoresis. Phylogenetic analysis revealed that all the samples clustered with *L. wolffii* within the P2 subclade, showing >99% shared identity with the *L. wolffii*-type strain Khorat-H2 (serovar Khorat) and those detected in Mymensingh in 2019 (Figure 2). Among the 22 samples, 16 samples formed a subcluster (BGD/2022/SC) with *L. wolffii* reported in other countries, e.g., KX245331 in Malaysia (2015) and EU497661 in Iran (2009).

The *Leptospira*-positive rates among the patients with fevers of variable duration were compared among the three detection methods (Table 1). IgM LAT and IgM ELISA showed the longest fever duration of 11–14 days (51–54%). In contrast, nested PCR exhibited a 54% positivity rate in 5–10 days, which was significantly higher than the rates observed using the other methods (36–38%). In the longer fevers (11 days or more), the positivity rates obtained using nested PCR were lower than those of IgM LAT and IgM ELISA.

The sociodemographic variables were compared between the leptospirosis and non-leptospirosis cases (Table 2). Leptospirosis was more commonly found in the age groups 16–30 years (38.6%) and 31–45 years (31.8%), but it was present in 17% of those aged 46–60 years, which was significantly lower than the rate among the *Leptospira*-negative patients. The rate of residents living in rural areas (80.7%) was higher among the cases of leptospirosis than that of those without the disease. Although no significant difference was detected in terms of the educational level, farmers accounted for 41% of the leptospirosis group, and the proportion of day laborers was significantly higher than that in the non-leptospirosis group. During the study period, both *Leptospira*-positive and -negative cases were the most common in May–June (44%), followed by March–April (28%).

The clinical symptoms and laboratory test values of the patients with and without leptospirosis are summarized in Table 3. Myalgia and jaundice showed a high incidence (>70%), while headaches and anorexia were also common (>56%). The incidence rates of jaundice, headaches, and oliguria in the leptospirosis group were significantly higher than those in the non-leptospirosis cases, likely associated with the increased serum bilirubin and creatinine levels. In addition, variable symptoms derived from the urinary and respiratory tracts, liver, and skin were found in <50% of the leptospirosis cases.

## 4. Discussion

Leptospirosis is an emerging zoonotic disease found all over the world and shows various clinical manifestations, ranging from asymptomatic/mild to severe illness. A small portion of patients have complications involving multiple organ systems, which results in a fatality ratio of >40% of cases [20]. Although timely diagnosis and treatment can reduce the severity of the illness, laboratory diagnostic tests are not always available, especially in low-income countries. Thus, this disease often remains undiagnosed or misdiagnosed as other febrile diseases. Recent reports documented the coinfection of leptospirosis and COVID-19 [21], and also dengue fever [22]. Bangladesh has also been affected by the COVID-19 pandemic, and more importantly, it has been experiencing serious dengue outbreaks yearly since 2018, with growing morbidity and mortality rates [23]. In such a situation, it may be possible that the prevalence of leptospirosis has been underestimated. Our study in north–central Bangladesh showed a considerably high prevalence of leptospirosis among the suspected patients, which was 47% (88/186) in the febrile patients, excluding those of known etiologic pathogens, although there is a possibility of selection bias because only patients who met the criteria were examined. This is comparable to the 48.9% described at the same study site in 2019 [16]. The previous studies in rural and urban areas in Bangladesh described a 8.4–38% prevalence of leptospirosis in febrile patients [12,13,14], while it was only 2% in four hospitals (Dhaka, Sylhet, and Rajshahi) in 2019–2020, just before the COVID-19 pandemic [17]. Thus, it is presumed that leptospirosis may be more prevalent in our study site, Mymensingh, which is a north–central region, than it is other regions studied in the country. The prevalence of serologically diagnosed leptospirosis in cases of febrile illness was reported to be 27–28% in northern and southern India [24,25], 8.4% in Malaysia [26], and 7.5% in northern Thailand [27], which were studied from 2000 to 2015. In African countries, its prevalence has been variable since 2000, but it has been described as less than 20% [28]. In northeast Thailand, the reduction in the prevalence of leptospirosis among patients with an acute undifferentiated fever was observed as 40% in 2001–2002 and 12.7% in 2011–2012 [29]. Considering these reports, leptospirosis seems to be endemic in Bangladesh, particularly at our study site, indicating the importance of its control, including accurate diagnosis and surveillance.

For the diagnosis of leptospirosis, several methods have been developed based on serological and indirect approaches, the direct identification of pathogens, or the detection of leptospiral genes [30]. In this study, we employed three methods to detect the antibodies and genes specific to *Leptospira* and found some difference in positivity depending on the method, although 63% of samples (55/88) were determined to be positive using all the methods. The discrepancy in the positivity rate using these methods was considered to be related to the duration of fever among the patients. Nested PCR exhibited a higher detection rate than IgM LAT and IgM ELISA among the patients with shorter fevers (<10 days), i.e., at an earlier stage of the disease. In contrast, the detection methods of IgM-diagnosed leptospirosis are more effective in the later stage of infection (fever of >11 days). This may be demonstrated by the finding that the antibody appears in the blood 5–7 days after infection, persisting for several months [31], while leptospires circulate in the blood mostly in the acute phase. Similarly to our study, the higher detection sensitivity of PCR was reported for serum samples in the early phase without detecting this antibody [32]. Therefore, it is recommended to use PCR in combination with serological tests, depending on the stage of the disease.

In this study, via the genetic analysis of the 16S rRNA, the species of all 22 samples were identified as *L. wolffii*, which belongs to the P2 subclade and was previously referred to as the pathogenically “intermediate” group [2,9]. This finding was also supported by the results of other genetic analyses (RFLP analysis and PCR with Lig1/Lig2 primers), according to which all the 78 samples were assigned to the “intermediate” group. Accordingly, it is suggested that *L. wolffii* was predominant in Mymensingh in 2022. Similarly, this species was the most common at the same study site in 2019 [16]. In contrast, in 2018, more *L. interrogans* was detected, with only one sample being assigned to *L. wolffii* [15], suggesting that the replacement of *L. wolffii* as the predominant species has occurred since 2019 in place of the previously common species *L. interrogans*. Although only limited information is available in Bangladesh, there is a report in 1992–1993 describing *copenhageni*, *australis,* and *icterohaemorrhagie* as dominant serovars [12], which belong to *L. interrogans* [10]. Another study of infection in rodents identified only pathogenic species, i.e., *L. interrogans*, *L. borgpetersenii,* and *L. kirschneri* [33].

*L. wolffii* was first described for the strain Khorat-H2 isolated from the urine of a patient in Thailand [34] and was previously classified into the intermediate or host-mediated pathogenic group; it causes mild or chronic disease in rare cases with unclear pathogenicity [2,9]. Thereafter, by means of the phylogenetic classification, *L. wolffii* was grouped into the P2 subclade, one of the two pathogenic groups (P1 and P2) [8] and revealed to have a similar virulence factor profile to that of the P1 subclade [35]. The P2 subclade is differentiated into three subgroups (P2-1, P2-2, and P2-3), among which *L. wolffii* is assigned to P2-2 as a sole member, having no other close species [11]. From human leptospirosis cases, *L. wolffii* has been detected as a minor species in Malaysia [36,37], India [38], Iran [39,40], and Argentina [41]. Furthermore, there are more studies documenting this species in cattle, rats, pigs, dogs, and wild animals in Asian countries [37,38,40,42] and Ecuador [43], as well as in environmental water and soil [44,45,46,47]. These findings suggest that *L. wolffii* may be more widely distributed among animals and also the environment, but it is rarely transmitted to humans. Accordingly, the high prevalence of *L. wolffii* and its presumptive persistence, as described in Mymensingh, Bangladesh, in our study, seem to be unusual. It is possible that the prevalence of *L. wolffii* might be associated with the increase in any host animal/reservoir harboring this species. To clarify this, the further surveillance of humans and animals may be necessary.

The characteristics of the patients and the symptoms of leptospirosis found in this study were generally similar to those reported previously in Bangladesh as well as worldwide [13,15,39,48,49]. This may imply that *L. wolffii*, the presumptive dominant species at this study site, has a similar pathogenicity in humans to those of the other pathogenic *Leptospira*. Although the high incidence rates of myalgia, headaches, and anorexia were similar to those reported in Bangladesh previously [13,15], significantly higher rates of jaundice and oliguria were associated with raised bilirubin and creatinine levels compared with those of the non-leptospirosis group. This may be attributed, in part, to the advanced stage of the disease, as shown by of the long fevers in many patients (>10 days for almost half of patients), or the putative elevated pathogenicity of *L. wolffii*. Thus, for better control of leptospirosis at our study site, we must clarify the reasons for such a late stage of disease, e.g., patients delaying hospital visits or medication consumption due to their low health literacy and/or economical condition and any virulence traits of *L. wolffii*. The bilirubin and creatinine levels in the leptospirosis group were significantly higher than those in the non-leptospirosis group, which may also characterize non-leptospirosis febrile diseases. Although the etiology of non-leptospirosis was not analyzed in this study, it is presumed that Rickettsial diseases may be one option because the presence of *R. felis* and *O. tsutsugamushi* had been described at the same study site [50,51].

This study indicated a currently high prevalence of leptospirosis in north–central Bangladesh, with the presumptive replacement of dominant *Leptospira* species in 2019. Because Bangladesh is located in a tropical monsoon region, its climate is characterized by high temperatures, heavy rainfall, and high humidity with seasonal variations [52]. The leptospirosis incidence increases one–two weeks after rainfall [53], as observed in our study, in which most cases were found in May–June, during the rainy season (May–September). In Thailand and Malaysia, leptospirosis had been prevalent during the 2010s, mostly in flooded areas or places associated with flooding [37,54]. Global climate change affects Bangladesh and causes extreme climate events, including rainfall and flooding [55], which may lead to an increase in the leptospirosis transmission risk via exposure to contaminated water and infected animals, particularly for populations with specific occupations [56]. Accordingly, systematic and continuous surveillance has become increasingly essential for the control of leptospirosis in this country.

## Figures and Tables

**Table 1:** Detection rates of Leptospira-specific IgM by LAT and ELISA, along with leptospiral DNA observed using nested PCR, in patients with fevers of variable duration.

Duration of Fever	Number of Samples	Number of Positive Sample (%)		
		IgM LAT	IgM ELISA	Nested PCR
5–10 days	81	29 (35.8%)	31 (38.3%)	44 (54.3%) *
11–15 days	67	36 (53.7%)	34 (50.8%)	32 (47.8%)
16–20 days	30	04 (13.3%)	04 (13.3%)	02 (6.7%)
>20 days	8	02 (25%)	0	0
Total	186	71 (38.2%)	69 (37.1%)	78 (41.9%)

* Significantly higher rate (*p* < 0.05) than those in other tests.

**Table 2:** Sociodemographic characteristics of patients with and without leptospirosis.

Sociodemographic Variables	Number of Cases (%)		
	Total (*n* = 186)	Leptospirosis (*n* = 88)	Non-Leptospirosis (*n* = 98)
Gender			
male/female	115 (61.8%)/ 71 (38.2%)	60 (68.2%)/ 28 (31.2%)	55 (56.1%)/ 43 (43.9%)
Age range			
0–15	3 (1.6%)	1 (1.1%)	2 (2.0%)
16–30	65 (34.9%)	34 (38.6%)	31 (31.6%)
31–45	54 (29.0%)	28 (31.8%)	26 (26.5%)
46–60	45 (24.2%)	15 (17.0%) *	30 (30.6%)
>61	19 (10.2%)	10 (11.4%)	9 (9.2%)
Locality			
Rural	130 (69.9%)	71 (80.7%) *	59 (60.2%)
Urban	56 (30.1%)	17 (19.3%)	39 (39.8%)
Educational level			
No education	36 (19.4%)	15 (17.0%)	21 (21.4%)
Primary education	70 (37.5%)	36 (40.9%)	34 (34.7%)
Secondary education	52 (28.0%)	25 (28.4%)	27 (27.6%)
Higher education	28 (15.1%)	12 (13.6%)	16 (16.3%)
Occupation			
Farmers	84 (45.2%)	36 (40.9%)	48 (49.0%)
Home-maker	28 (15.1%)	15 (17.0%)	13 (13.3%)
Day laborer	26 (14.0%)	18 (20.5%) *	8 (8.2%)
Student	19 (10.2%)	8 (9.1%)	11 (11.2%)
Others	29 (15.6%)	11 (12.5%)	18 (18.4%)
Seasonal variation			
November–December	25 (13.4%)	13 (14.8%)	12 (12.2%)
January–February	22 (11.8%)	11 (12.5%)	11 (11.2%)
March–April	50 (26.9%)	25 (28.4%)	25 (25.5%)
May–June	89 (47.8%)	39 (44.3%)	50 (51.0%)

* *p* < 0.05.

**Table 3:** Clinical characteristics of patients with and without leptospirosis.

Clinical Characteristics	Number of Cases (%)	
	Leptospirosis (*n* = 88)	Non-Leptospirosis (*n* = 98)
Symptoms		
Fever	88 (100%)	98 (100%)
Myalgia	74 (84.1%)	70 (71.4%)
Jaundice	62 (70.5%) *	55 (56.1%)
Headache	55 (62.5%) *	40 (40.8%)
Anorexia	50 (56.8%)	42 (42.9%)
Abdominal pain	34 (38.6%)	30 (30.6%)
Cough	30 (34.1%)	24 (24.5%)
Oliguria	28 (31.8%) *	17 (17.3%)
Hepato-splenomegaly	18 (20.5%)	14 (14.3%)
Conjunctival suffusion	14 (15.9%)	8 (8.2%)
Skin rash	11 (12.5%)	8 (8.2%)
Sign of meningitis	4 (4.5%)	2 (2.0%)
Laboratory findings		
Raised serum bilirubin (>1 mg/dL)	62 (70.5%) *	50 (51.0%)
Raised serum creatinine (>1.2 mg/dL)	30 (34.1%) *	12 (12.2%)
Thrombocytopenia (< 150,000/mL)	22 (25.0%)	15 (15.3%)
Raised ALT (>40 IU/L)	45 (51.1%)	38 (38.8%)
Raised AST (>38 IU/L)	38 (43.2%)	32 (32.7%)
Leukocytosis (>11,000/mm^3^)	35 (39.8%)	30 (30.6%)
Proteinuria (>150 mg/day)	22 (25.0%)	15 (15.3%)

ALT = Alanine Transaminase; AST = Aspartate Transaminase; * Significantly higher rate (*p* < 0.05) than non-leptospirosis group.

## Data Availability

In this manuscript, all the data were described in text, as well as GenBank accession numbers.
